# Expression of PD-L1 Is Associated with Inflammatory Microenvironment in Surgical Specimens of Non-Small Cell Lung Cancer

**DOI:** 10.3390/jpm11080767

**Published:** 2021-08-04

**Authors:** Ivan Simundza, Dragan Krnic, Josko Juricic, Benjamin Benzon, Rina Simundza, Ivan Mario Stanicic, Vesna Capkun, Katarina Vukojevic, Merica Glavina Durdov

**Affiliations:** 1 Department of Surgery, University Hospital of Split, 21000 Split, Croatia; isimundza@kbsplit.hr (I.S.); drkrnic@gmail.com (D.K.); joskojuricic123@gmail.com (J.J.); 2 Department of Anatomy, Histology and Embryology, School of Medicine, University of Split, 21000 Split, Croatia; benjamin.benzon@mefst.hr (B.B.); vesna.capkun@mefst.hr (V.C.); 3 Department of Gynaecology, University Hospital of Split, 21000 Split, Croatia; rinapejkovic@gmail.com; 4 Department of Pathology, University Hospital of Split, 21000 Split, Croatia; istanicic@kbsplit.hr

**Keywords:** PD-L1, NSCLC, lung surgery, overall survival, immunotherapy

## Abstract

The aim of this study was to analyse the expression of PD-L1 in non-small cell lung cancer (NSCLC) and its correlation with immune microenvironment response (IMR), clinic-pathological parameters, and outcome. The sample included 76 male and 32 female patients who underwent surgical resection. The mean age of the males was 66 years, and that of the females was 64 years. Adenocarcinoma (ADC) was diagnosed in 68 (63%) cases, squamous cell carcinoma in 35 (32%) cases, and NSCLC (not otherwise specified) in 5 (5%) cases. Metastatic lymph nodes were found in 38 (36%) patients, 18 with N1 nodes and 20 with N2 nodes. PD-L1 expression was valuated as the percentage of positive cancer cells among all cancer cells. Gender, age, and histologic type were not associated with PD-L1 expression (all *p* > 0.05). The subtypes of ADC were associated with PD-L1 expression (*p* = 0.050). The papillary subtype was 4.3 times more common among PD-L1 negative than PD-L1 positive ADC; the solid subtype was 1.9 times more common among PD-L1 positive than PD-L1 negative ADC. IMR was predominantly strong in 19 cases, weak in 36, and absent in 53 cases. The median value of PD-L1 expression in cancer cells was positively correlated with IMR (*p* = 0.039). PD-L1 expression was not correlated with overall survival (*p* = 0.643). The patients with strong, inflammatory-like IMR had an average survival time that was 12 months longer than patients with absent/low IMR (LR = 2.8; *p* = 0.132). In conclusion, the papillary subtype was more commonly PD-L1 negative in comparison with other subtypes of ADC. Positive PD-L1 expression in tumour cells was connected with strong, inflammatory-like IMR. Patients with strong IMR tended to experience better outcomes. Further investigations are needed on larger-scale cohorts to elucidate the insights of this descriptive study.

## 1. Introduction

Lung cancer (LC) is the most common cause of cancer death [1]. In the last decade, classic chemo-radiotherapy for metastatic non-small cell lung cancer (NSCLC) has given way to a new paradigm of personalised therapy. The high mortality rate of LC in Croatia encouraged the adoption of the National Screening program of early detection by low-dose computed tomography, which started in 2020 [1]. One of the goals is to diagnose LC in the early stages when the surgical procedure is the treatment of choice. In the postoperative follow-up, disease-free survival was different among the patients, although most of them died of lung cancer. A small biopsy is a source of diagnostic and predictive data and reflexing testing for tyrosine kinase inhibitors, and immune checkpoint molecules PD-1/PD-L1 become routine in diagnostic practice [2]. PD1/PD-L1 interaction enables tumour cells to escape from the host immune response and is important for cancer progression [3,4]. Therefore, today, PD1/PD-L1 and their inhibitors are a focus of clinical interest for application in the therapy of most common human cancers [3,5,6]. The immunotherapy blocks the PD-1/PD-L1 signalling pathway and thus recovers antitumor immunity. Several anti-PD-1/PD-L1 inhibiting antibodies have been introduced as a second-line therapy for patients with advanced NSCLC or as a first-line therapy combined with chemotherapy for patients with squamous cell carcinoma (SCC) [7]. Pembrolizumab is a humanised monoclonal antibody against a programmed death-1 protein that blocks its interaction with PD-L1 and PD-L2 [8]. For the early stages, curative surgery with adjuvant chemotherapy/immunotherapy is the main therapeutic option. For the assessment of the PD-L1 status in a small biopsy, the percentage of positive PD-L1 cancer cells (<1%, 1–49%, >49%) among at least 100 cancer cells is determined [9]. We wanted to analyse the native expression of PD-L1 in primary cancer and metastatic lymph nodes and compare it to clinic-pathological parameters. PD-L1 analysis on surgical material provides us with the opportunity to see the heterogeneity of PD-L1 expression in the native tumour milieu. We analysed the expression of PD-L1 in primary cancer and metastatic lymph nodes of patients with operable NSCLC who were therapeutically naive. The aim of the study was to analyse the expression of PD-L1 in cancer cells in relation to the tumour microenvironment, clinic-pathological parameters, and outcome in the era before lung cancer immunotherapy.

## 2. Materials and Methods

This retrospective study included 108 patients with NSCLC who underwent surgery from 2012 to 2018 at the Department of Surgery, University Hospital in Split, Croatia. Clinical data were collected from the hospital records, and paraffin blocks were collected from the archives of the Department of Pathology. Patients with NSCLC with complete clinical data and paraffin block of tumour tissue were included in the study. Exclusion criteria were atypical resection, another type of cancer, lung metastases, or incomplete follow-up data. The follow-up of the patients was 53 months, starting from the time of diagnosis. Overall survival (OS) was calculated from the date of diagnosis to the date of death from any cause or was censored at the last follow-up date, 1 January 2019. The study was conducted in accordance with the provisions of the Declaration of Helsinki and was approved by the Ethics committee of the University Hospital in Split (number 500-03/18-01/18).

**Figure 1:**
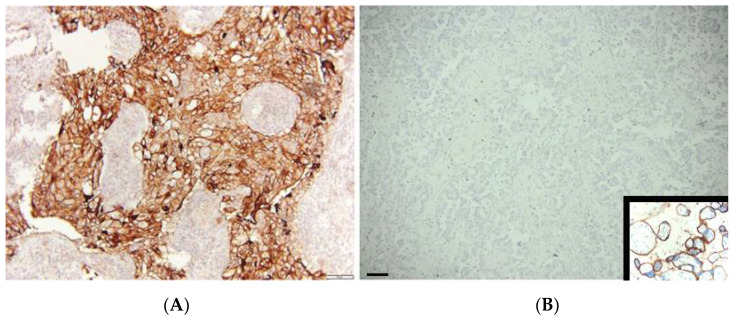
PD-L1 expression in cancer cells of NSCLC (**A**) strongly positive and (**B**) completely negative. The inset on panel B represents the PD-L1 positive trophoblast cells in placental villi. Scale bar 25 µm.

Five µm thick slides were cut from the paraffin blocks and pretreated in a standard way. The immunostaining was performed on Ultra Benchmark (Ventana Medical Systems, Inc. Oro Valley, AZ, USA) using rabbit polyclonal antibody PD-L1 (22C3 pharm DX; DAKO, Carpenteria, CA, USA) and according to the manufacturer instructions. Additionally, in order to determine the inflammatory microenvironment, mouse monoclonal antibody CD8 (Ventana Medical Systems, Inc., Oro Valley, AZ, USA) was used. PD-L1 antibody is approved by the FDA as a companion diagnostic for pembrolizumab therapy. Microscopic analysis was performed on the light microscope Olympus BX41 (Olympus, Tokio, Japan) by two independent pathologists. Five high-power magnification fields (400×) were analysed. Positive cancer cells display the complete circumferential or partial cell membrane staining of any intensity (Figure 1). The percentage of PD-L1 positive cancer cells among the total tumour cells was estimated as a tumour proportion score, TPS. Tumour-associated immune cells, such as macrophages, were excluded from scoring. All cancers that showed TPS ≥ 1% were considered PD-L1 positive [10]. In the metastatic lymph nodes, the PD-L1 status of the tumour was estimated positive if any cancer cell expressed PD-L1. The external positive control was trophoblast cells in the human placenta. The external negative control was staining without the application of a primary antibody.

**Figure 2:**
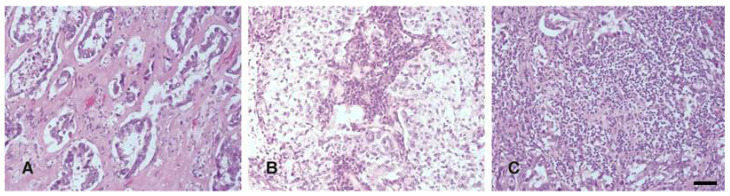
Immune microenvironment response in the tumour: predominantly absent (**A**), low (**B**), or high (**C**). Scale bar 25 µm.

IMR was analysed on middle-power magnification fields (200×) on the original HE slides of the primary cancer (the centre and the margin). According to the number of immune cells (few, mild, or high) and the pattern of tumour stroma (desmoplastic or sparse), IMR was qualified as absent (few immune cells in the desmoplastic stroma), low (mild number of immune cells in the broad front to clusters of tumour cells in the desmoplastic stroma), and strong (very dense infiltration of immune cells mixed with the tumour in the sparse stroma) (Figure 2).

**Figure 3:**
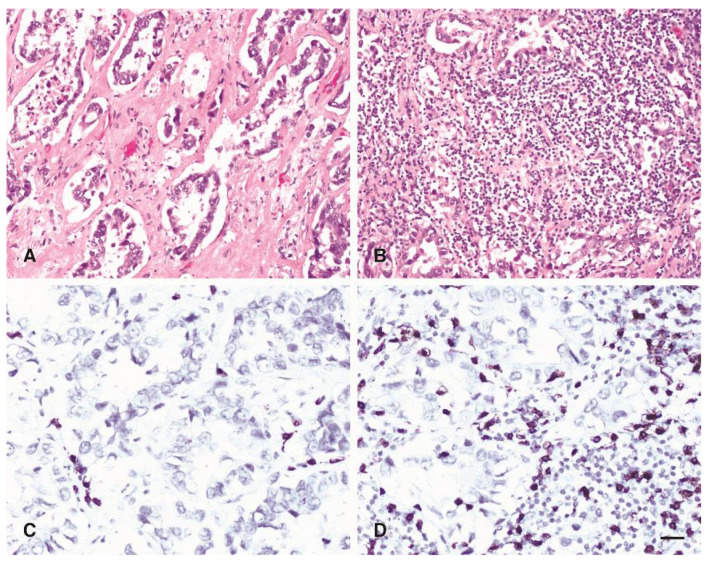
Tumour microenvironment response: (**A**,**C**) low with few CD8 + cytotoxic lymphocytes, and (**B**,**D**) high (inflammatory) with numerous CD8+ cytotoxic lymphocytes. Scale bar 25 µm.

Additionally, IMR was confirmed by CD8 immunostaining (Figure 3).

In the statistical processing of the qualitative data, the χ^2^ test and Fisher’s Exact Test were used. The Kruskal–Wallis test was used to compare the quantitative variables between the three observed groups. The Mann–Whitney U test was used to compare the quantitative variables between the two groups, the Kaplan–Meier curve was used to show OS, and the Log Rank test was used to compare survival. The SPSS 20 statistical package was used for data processing. A *p*-value of <0.05 was considered statistically significant in all tests.

## 3. Results

The sample included 76 males and 32 females. The median age of the males was 66 years (Q1–Q3: 60–73; min–max: 50–84), and that of the females was 64.5 years (Q1–Q3: 58–70; min-max: 45–76) (Z = 1.23; *p* = 0.219). The TNM stages of patients were determined according to IASLC criteria [11]. Postoperative pathological stages (*p*) were: *p* I in 51 (47%), *p* II in 31 (29%), and *p* III in 26 (24%) patients. ADC was diagnosed in 68 (63%), SCC in 35 (32%), and NSCLC-NOS in 5 (5%) patients. Metastatic lymph nodes were found in 38 (35%) patients: 18 in the peripheral or hilar/interlobular zone (N1 nodes) and 20 in the mediastinal region (N2 nodes). PD-L1 expression in tumour cells was completely negative in 61 cases, positive in 1–49% in 29 cases, and positive in ≥50% in 18 cases. The distribution of patients according to analysed variables and TPS 0 or ≥1 is presented in Table 1.

**Figure 4:**
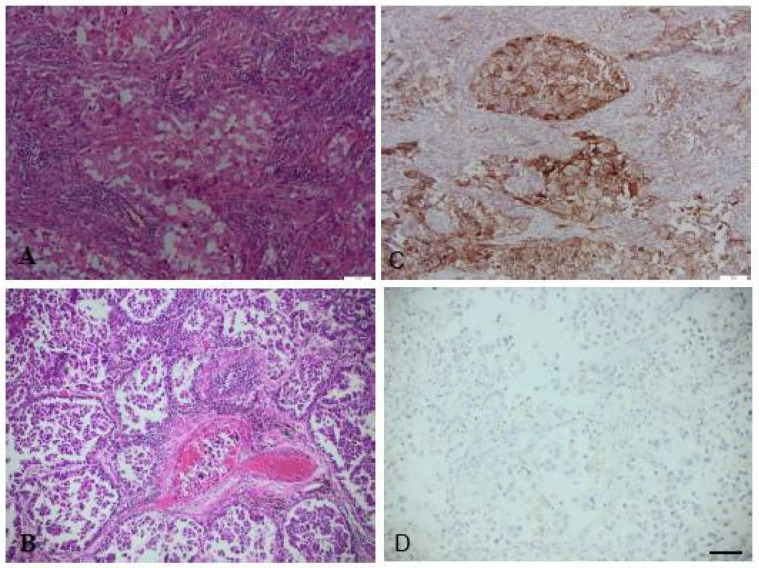
In ADC (**A**—hematoxylin and eosin staining), positive PD-L1 expression is more common in solid subtype—brown staining (**C**) while in the papillary subtype (**B**—hematoxylin and eosin staining), PD-L1 expression in the papillary subtype is negative (**D**). Scale bar 25 µm.

The distribution of patients by sex (χ^2^ = 0; *p* = 1), grade (χ^2^ = 3.83; *p* = 0.147), histological type (χ^2^ = 0.612; *p* = 0.434), the presence of atelectasis/pneumonitis (χ^2^ = 2.65; *p* = 0.104), the presence of lympho-vascular invasion (χ^2^ = 1.42; *p* = 0.233), and disease stage (χ^2^ = 2.3; *p* = 0.320) did not differ statistically according to negative or positive PD-L1 expression. There was no significant difference in age (Z = 1.1; *p* = 0.268) and tumour size (Z = 1.84, *p* = 0.066) among patients with negative or positive PD-L1 expression. The distribution of the ADC subtype was different according to the expression of PD-L1 (χ^2^ = 5.98; *p* = 0.050). There were 4.3 times more patients with the papillary subtype in PD-L1 negative tumours than in PD-L1 positive tumours (Figure 4). The distribution of acinar and papillary subtype according to PD-L1 status was different at the significance level of 95% (*p* = 0.053, Fisher’s exact test). The chance ratio for PD-L1 negative tumours in patients with papillary subtype was 5.1 times higher than for acinar tumours (OR = 5.1; 95% CI: 0.999-26; *p* = 0.050). The distribution of solid and papillary subtype according to PD-L1 status was different (*p* = 0.046, Fisher’s exact test). The probability ratio for PD-L1 negative tumours in patients with papillary subtype was 8 times higher than for solid tumours (OR = 8; 95% CI: 1.3–50; *p* = 0.026). We did not find a statistically significant difference between the distribution of acinar and solid adenocarcinoma subtype according to the expression of PD-L1 (*p* = 0.541, Fisher’s exact test).

In 19 cases, IMR was predominantly strong; in 53 cases, IMR was low; and in 53 cases, IMR was predominantly absent. There was a statistically significant difference in the median expression value for PD-L1 (%) in the IMR groups (χ^2^ = 6.5; *p* = 0.039). It was higher in the group with strong IMR than in the groups with absent IMR and low IMR (*p* = 0.043, pairwise test). There were differences in the median expression value for PD-L1 in three ADC subtypes (χ^2^ = 8.5; *p* = 0.014). There was only a statistically significant difference between the solid subtype and the papillary subtype (*p* = 0.011; pairwise test) (Table 2).

Among 108 patients, 38 had positive lymph nodes. The association between PD-L1 expression in primary tumours and nodal metastasis is presented in Table 3. There was no statistically significant difference in TPS, as well as in the median expression value for PD-L1 in primary tumours and nodal metastasis (Z = 0.421; *p* = 0.673).

The average OS for 108 patients was 48.7 months (SE: 3.5; 95% CI: 42–55); the median was 48 months (SE: 8; 95% CI: 42–55). The OS was calculated for each examined variable (Table 4). A significant difference in OS was found according to lympho-vascular invasion (*p* = 0.012) and ADC subtype (*p*= 0.009). The median OS was 20 months longer for patients without lympho-vascular invasion than for patients with lympho-vascular invasion. The median OS in the solid subtype was 31 months shorter than in the papillary subtype and 41 months shorter than in the acinar subtype. Patients with absent/low IMR had no difference in OS (LR = 0.445; *p* = 0.505).

The results of the univariate Cox’s test are shown in Table 5. ADC subtype (*p* = 0.016) and lympho-vascular invasion (*p* = 0.015) were significantly associated with a shorter OS. The HR of fatal outcomes was 4.2 times higher in solid than in acinar subtype (*p* = 0.004). The HR of fatal outcomes was 2.15 times higher for patients with lympho-vascular invasion than for patients without lympho-vascular invasion (*p* = 0.015). Patients with absent/low IMR had 2 times higher HR than patients with strong IMR at the significance level of 90% (*p* = 0.142).

Table 6 shows the distribution of 47 patients who died in the follow-up period, according to the length of OS: ≤24 months and >24 months. During the first 24 months, 29 patients died. The median of their OS was 10 months (Q1–Q3: 6–16; min–max: 1–24). In total, 18 patients demised after 24 months. The median of their OS was 37 months (Q1–Q3 = 32–50; min-max: 27–68 months). There was no statistically significant difference between patients who lived ≤24 months and patients who lived >24 months according to analysed variables (*p* > 0.05). Among those who demised, five patients had inflammatory TMR, and all lived longer than 24 months (data not shown).

## 4. Discussion

The main finding of our study is the correlation between the median PD-L1 expression values and the subtypes of ADC and IMR, consecutively. Namely, the papillary subtype of ADC is more often PD-L1 negative than the other subtypes. Similar to our study, Miyatawa et al. found PD-L1 negativity in the papillary subtype of ADC [12]. A possible explanation is that the growth pattern of the solid and acinar subtype has a wider contact with the immune microenvironment than the papillary subtype that grows partially in the alveolar spaces. Immune cells in the microenvironment are likely to induce PD-L1 on tumour cells. JAK-STAT signalling is important to PD-L1 regulation in response to inflammatory cytokines, such as interferon gamma [13]. According to Tancoš et al., PD-L1 is associated with an inflammatory background, measured by CD8 + lymphocytes in the microenvironment [14]. Likewise, Lin et al. published similar results [10]. Inoue et al. described the high intensity of immune infiltrates as the pathological factor that predicts PD/L1 positivity [15]. We did not find a difference in PD-L1 expression between ADC and SSC. According to Muller et al., ADC shows increased signalling through TYK2, which transmits signalling through interferons and has more phosphorylation of STAT3 than SCCs. SCCs have less TYK2 and p-STAT3 due to increased proteasomal degradation of TYK2 by an E3 ubiquitin ligase called SIAH2 [16].

On the other side, the mutation of PDL-l as amplification is an autonomous signal to the tumour immunosurveillance. This could imply that a positive correlation between IMR and PD-L1 expression was found in our study. In the literature, the connection between PD-L1 and OS is controversial [13]. However, we did not find a significant correlation between OS and PD-L1. Pawelczyk et al. showed that PD-L1 expression seems to be associated with increased tumour proliferation and aggressiveness, as well as shorter patient survival in NSCLC, predominantly in the ADC [17]. This difference might be due to the smaller number of cases in our study (108 in contrast with 800) or the different techniques employed (primary sample in contrast with tissue microarray). We did not find any differences between the expression of PD-L1 in primary tumours and nodal metastasis. In a comparative analysis between primary tumours and synchronous regional lymph node metastases, Inoue et al. revealed that the PD-L1 gene copy number alterations were highly consistent and reproducible compared with the PD-L1 expression [15]. We found a correlation between high (inflammatory-like) IMR and PD-L1 expression. This might imply the importance of IMR in a native tumour setting as a predictive factor itself and in combination with the PD-L status. In their study, Inoue et al. [15] found that the high intensity of tumour infiltrate is connected to the PD-1 expression but not to the PD-L1 copy number status. It is well known that tumour mutation burden, the expression of PD-L1, and immune cells infiltration reflect upon immune response and survival [18]. So far, there have been some studies on LC that revealed similar findings [19]. In our study, the patients with high (inflammatory) IMR had the longest average survival (12 months longer than patients with absent/low IMR). In the study, we divided 47 patients who died in the follow-up period into two groups according to the length of OS: ≤24 months and >24 months. In total, 29 patients died in a period shorter than 2 years, and 18 patients lived more than 2 years. We did not find any significant differences in the analysed variables between the groups. It is interesting to note that only 5 out of the 47 patients who died had strong (inflammatory) IMR, and all of them were in the group who lived longer than 2 years. This could potentially imply that the inflammatory response pattern in NSCLC has better a prognosis in terms of OS compared to the low immune response patterns. In our study, patients with a solid predominant subtype of ADC were observed in relation to the acinar and papillary subtype, and the risk of death was found to be 1.84 times higher than other ADC subtypes. Similar to our results, Zhang et al. showed that the solid subtype is an independent poor prognostic factor and an independent negative predictor for patients with lung ADC [20]. The risk of death was also 2.15 times higher for patients with lympho-vascular invasion compared to patients without it. Mediastinal lymph node status is known as one of the strongest single independent prognostic factors for patients with NSCLC [21]. In our study, we showed that the risk of death for the group of patients with positive mediastinal lymph nodes (N2 disease) was 1.86 times higher than for others. Although our study about PD-L1 expression used archival material, we presume its clinical relevance. Namely, according to Herbst et al., no significant difference exists between newly collected and archival material [22].

The limitations of this study are the small sample, the descriptive character of the study, and the classical pathological analysis instead of the technical novelty. An artificial intelligence functional analysis of the tissue would considerably improve our manuscript; however, we are still not able to use these advanced methods at our institution. Additionally, we did not have any information on patient smoking history. The advantages are that the study is based on primary samples of LC patients who underwent surgery before the introduction of immunotherapy in Croatia and who were therapeutically naive.

In conclusion, according to our study, PD-L1 expression in tumour cells was associated with IMR. Furthermore, the papillary subtype is more often negative for PD-L1 compared to other ADC subtypes. High (inflammatory) IMR is more favourable for the patient’s OS than absent/low IMR. These results should be verified in larger studies involving patients and their clinical data.

## Figures and Tables

**Table 1:** The correlation of analysed variables with PD-L1 expression.

VARIABLES		PD-L1 Tumour Proportion Score		
		0	≥1	*p*
Gender; n (%)				
Males	76 (70)	43 (70)	33 (70)	1 *
Females	32 (30)	18 (30)	14 (30)	
Age; median (Q1–Q3; min–max)		65 (58–71; 45–82)	66 (61–73; 51–84)	0.268 *
NSCLC; n (%)				
ADC	68 (63)	40 (67)	28 (58)	0.434 *
SCC	35 (32)	17 (28)	18 (38)	
NSCLC-NOS	5 (5)	3 (5)	2 (4)	
Tumour size; median (Q1–Q3; min–max)		3 (2–4.5; 1–15)	3 (2–4; 1–10)	0.066 **
Gradus; n (%)				
1	15 (14.2)	11 (18.6)	4 (8.5)	0.147*
2	59 (55.7)	34 (57.6)	25 (53.2)	
3	32 (30.2)	14 (23.7)	18 (38.3)	
Atelectasis or pneumonitis; n (%)				
Yes	49 (45)	23 (38)	38 (62)	0.104 *
No	59 (55)	26 (55)	21 (45)	
Lympho-vascular invasion; n (%)				
No	47 (44)	23 (38)	24 (51)	0.233 *
Yes	61 (56)	38 (62)	23 (49)	
Stage; n (%)				
1	51 (47.2)	27 (44.3)	24 (51.1)	0.320 *
2	31 (28.7)	16 (26.2)	15 (31.9)	
3	26 (24.1)	18 (29.5)	8 (17)	
ADC subtype; n (%)				
Acinar	37 (56.9)	20 (52.6)	17 (63)	0.050 *
Papillary	14 (21.5)	12 (31.6)	2 (7.4)	
Solid	14 (21.5)	6 (15.8)	8 (29.6)	
ADC subtype; n (%)				
Acinar	37 (72)	20 (62)	17 (89)	0.053 †
Papillary	14 (28)	12 (38)	2 (11)	
ADC subtype; n (%)				
Papillary	14 (50)	12 (67)	2 (20)	0.046 †
Solid	14 (50)	6 (33)	8 (80)	

* χ^2^ test; ** Mann–Whitney U test; † Fisher’s Exact Test. Note: only three patients with ADC had lepidic subtype and were excluded from the analysis.

**Table 2:** The correlation between analysed variables and the median value for PD-L1 expression.

		Median Expression Value for PD-L1 (%) (Q1–Q3; Min–Max)	*p*
Histological type			
	ADC	0 (0–20; 0–100)	0.699 *
	SCC	1 (0–25; 0–90)	
Lympho-vascular invasion			
	No	1 (0–50; 0–100)	0.95 *
	Yes	0 (0–4; 0–100)	
Lymph node status			
	Negative	0 (0–25; 0–100)	0.705 *
	Positive	0 (0–5; 0–100)	
Pathological stage			
	1	0 (0–25; 0–100)	0.505 **
	2	0 (0–5; 0–90)	
	3	0 (0–5,7; 0–100)	
IMR			
	Absent	0 (0–1; 0–90)	0.039 **
	Low	0 (0–25; 0–100)	
	Strong	3 (0–50; 0–100)	
Histological grade			
	1	0 (0–1; 0–60)	0.119 *
	2	0 (0–5; 0–100)	
	3	1 (0–50; 0–100)	
ADC subtype			
	Acinar	0 (0–12,5; 0–60)	0.014 **
	Papillary	0 (0–0; 0–20)	
	Solid	25.5 (0–90; 0–100)	

* Mann–Whitney U test, ** Kruskal–Wallis test. IMR immune microenvironment response.

**Table 3:** The correlation between PD-L1 expression in cancer cells in primary tumours and metastatic lymph nodes.

Primary Tumour	All	PD-L1 in Lymph Node		*p*
		Negative (0)	Positive (>0)	
Expression of PD-L1; median (Q1–Q3; min–max)	0 (0–19; 0–100)	0 (0–60; 0–100)	0 (0–4.5; 0–90)	0.673 *
PD-L1; n (%)				
negative	22 (58)	9 (70)	13 (52)	1 **
positive	16 (42)	4 (30)	12 (48)	

* Mann–Whitney U test, ** Fisher’s exact test.

**Table 4:** Overall survival of patients with surgically treated NSCLC according to analysed variables.

Variable	OS (Months)	SE	95% CI	Median	LR	*p*
Gender						
Males	46	3.9	38–53	44	1.65	0.199
Females	50	5	41–60			
Histologic type						
ADC	49.8	3.9	42.2–57.5	62	2.14	0.143
SCC	42.4	5.6	31.4–53.5	38		
Adenocarcinoma subtype						
Acinar	59	4.6	50–68	68		
Papillary	43.5	7.8	28–59	37	4.5	0.009
Solid	24.8	3	18.9–31	27		
Histological gradus						
1	51	6.6	38–64	62	1.07	0.585
2	47.2	4.2	39–55	48		
3	45.2	6.6	32–58	34		
Lympho-vascular invasion						
No	58	5	48–68	68	6.3	0.012
Yes	38.8	4	31–47	37		
Pathological stage						
1	52.4	4.4	44–61	62	2.99	0.224
2	44	6	33–56	38		
3	38	5.5	27–49	37		
Lymph node status						
Negative	51	4	42–59	53	1.4	0.235
Positive	41	5	31–51	37		
PD-L1						
Negative	47.9	4.5	39–57	44	0.15	0.643
Positive	47	4.7	38–56	48		
IMR						
Absent	43.5	4.7	34–53	37	2.8	0.246
Low	46	5	36–56	49		
Strong	58	6	45–71			

Log rank test. IMR immune microenvironment response.

**Table 5:** Cox regression uninominal analysis of OS according to the examined variables.

	HR	95% CI	*p*
Sex			
Male *	0.636	0.32–1.28	0.206
Female			
Histological type			
ADC *	1.3	0.8–2.02	0.304
SSC			
ADC subtype			
Acinar *			0.016
Papillary	1.96	0.7–5.5	0.203
Solid	4.2	1.6–11.4	0.004
Gradus			
1 *	1.26	0.8–1.97	0.313
2			
3			
Lympho-vascular invasion			
No *	2.15	1.17–3.98	0.015
Yes			
Pathological stage			
1 *	1.33	0.94–1.9	0.108
2			
3			
Lymph node status			
Negative *	1.4	0.79–2.5	0.241
Positive			
PD-L1 expression			
Negative *	0.87	0.48–1.6	0.646
Positive			
IMR			
Absent + Low	2	0.79–5	0.142
Strong *			

M—male; F—female; * level of reference IMR immune microenvironment response.

**Table 6:** Dead patients (n = 47) according to analysed variables and OS (≤24 months; >24 months).

		Lived (Months)		
		≤24	>24	*p* *
Lymph node status; n (%)				
Negative	28 (60%)	16 (55%)	12 (67%)	0.635
Positive	19 (40%)	13 (45%)	6 (33%)	
PD-L1; n (%)				
Negative	29 (62%)	20 (69%)	9 (50%)	0.321
Positive	18 (38%)	9 (31%)	9 (50%)	
Gradus; n (%)				
1	6 (13%)	2 (7%)	4 (22%)	0.190
2	24 (32%)	14 (50%)	10 (56%)	
3	16 (35%)	12 (43%)	4 (22%)	
Lympho-vascular invasion; n (%)				
No	16 (34%)	9 (31%)	7 (39%)	0.814
Yes	31 (66%)	20 (69%)	11 (61%)	
Histological type; n (%)				
ADC	25 (56%)	16 (57%)	9 (53%)	1
SCC	20 (44%)	12 (43%)	8 (47%)	
Subtype of ADC; n (%)				
Acinar	9 (37.5%)	5 (33.3%)	4 (44.4%)	small sample size
Papillary	6 (25%)	4 (26.7%)	2 (22.2%)	
Solid	9 (37.5%)	6 (40%)	3 (33.3%)	

* χ^2^ test.

## Data Availability

Not applicable.
